# Editorial: Online User Behavior and User-Generated Content

**DOI:** 10.3389/fpsyg.2022.895467

**Published:** 2022-04-25

**Authors:** Jose Ramon Saura, Yogesh K. Dwivedi, Daniel Palacios-Marqués

**Affiliations:** ^1^Department of Business Economics, Rey Juan Carlos University, Madrid, Spain; ^2^School of Management, Swansea University, Wales, United Kingdom; ^3^Symbiosis Institute of Business Management, Symbiosis International (Deemed University), Pune, India; ^4^Department of Business Organization, Universitat Politècnica de València, Valencia, Spain

**Keywords:** online user behavior, user-generated content, artificial intelligence, digital marketing, social networks, privacy, UGC, LDA

Research on online user behavior and what is known as user generated-content (UGC) has become a key element for the effective development of digital strategies (Kar and Dwivedi, 2020; Lakshmi and Bahli, 2020; Reyes-Menendez et al., 2020). The increase in the use of social networks and the Internet, both by users and companies, has resulted in new data points being constantly generated between the behaviors and actions of online users when they interact with advertising elements that are part of companies' digital marketing strategies (Ribeiro-Navarrete et al., 2021).

In this digital ecosystem, the use of new technologies for the analysis of behavioral data and UGC has grown exponentially (Martín and Fernández, 2022). Accordingly, it is of vital importance to understand user experience, their opinions, digital actions, customer journeys, and browsing habits, among others, so that to obtain analytical indicators that help predict their actions, increase the profitability of digital advertising, and make better decisions.

The emergence and increase of artificial intelligence (AI) techniques applied to data analysis, both as concerns user behavior and UGC production, allows companies to predict user behavior and identify patterns. With these techniques, insights can be extracted and original knowledge can be identified that helps companies establish robust and profitable digital strategies. However, concerning this digital paradigm, there have been concerns about the privacy of users in relation to the treatment and collection of their data, behavior, and prediction of their actions (Adamides and Karacapilidis, 2020; Akter et al., 2021; Dwivedi et al., 2021).

The use and application of AI has increased the development of data-driven models (Saura et al., 2021). Such models include algorithmic systems that, through innovation in the analysis of user data, are capable of predicting their behavior and personalizing the content in social networks or on digital platforms, among other channels. Consequently, personalization of content in real time considerably increases the profitability of digital marketing actions.

As argued by Saura et al., 2021, the application of algorithms that work with machine learning is a key element to understand how user behave online. In this context, Ding et al. show in their article the use of two data-driven models for the analysis of UGC datasets. On the one hand, the authors apply a sentiment analysis algorithm that identifies the sentiments of the UGC sample of Airbnb users and a well-known topic modeling algorithm called Latent Dirichlet allocation (LDA), which is used to identify topics in a specific sample. In their analysis of different sources of satisfaction when using the Airbnb platform, the authors show the application of original approaches to identify insights that can improve decision making in digital ecosystems and transform UGC data and behavior into useful knowledge to improve consumer satisfaction and evaluate new management implications.

In another contribution to this Research Topic, Zloteanu et al. link the study of UGC with the theoretical framework of trust and reputation information (TRI) analysis to understand the role of user judgment and decision making in UGC. The authors identify insights and propose creating original knowledge linked to the sharing economy (SE). In addition, the authors argue that the study of users and their content can modify SE judgments in digital channels. Accordingly, research on how users behave in digital environments is understood as being able to measure and control both online reputation and trust in digital environments in relation to a social movement, event, or membership in a community, among others.

Martínez-Navalón et al. discuss the relevance of the analysis of user behavior through the theoretical framework of electronic Word of Mouth (e-WOM) where the identification of how users interact with each other or with elements of social networks (engagement) is a fundamental value for the study of the generation of UGC (Siddique et al., 2021). Specifically, the authors use a Partial Least Squares Structural Equation Modeling (PLS-SEM) model to measure user trust and satisfaction on the TripAdvisor platform. This contribution reveals a positive relationship between the perception of privacy and user satisfaction on the digital platform.

In another contribution, Xu et al. explore user willingness to pay for online knowledge, considering the associations that exist between the UGC, online user behavior, and user attitudes to make purchases in digital environments and social networks. To this end, using a model developed in PLS-SEM, the sentiment of belonging to social networks and digital communities is linked to perceived risk and group conformity, and these variables are found to indirectly affect consumers' willingness to pay in digital environments. These insights highlight the relevance of studying online user behavior and their attitudes that could be predicted by AI algorithms.

Finally, this Research Topic offers insights to specifically understand online user behavior, UGC, and strategies that use AI to extract knowledge in a new and original way. In addition, models and approaches are identified to better understand a complex ecosystem where respect for data privacy and the application of data-driven models to make predictions are becoming increasingly important to gain ethical profitability in digital marketing and social networking strategies. In this way, the contributions published in this Research Topic identify gaps and propose future lines of research, strengthening understanding of the challenges faced by this research field.

## Author Contributions

JS drafted this contribution. YD and DP-M revised and modified this contribution. All authors contributed to the article and approved the submitted version.

## Funding

In gratitude to the Ministry of Science, Innovation and Universities and the European Regional Development. Fund: RTI2018-096295-B-C22.

## Conflict of Interest

The authors declare that the research was conducted in the absence of any commercial or financial relationships that could be construed as a potential conflict of interest.

## Publisher's Note

All claims expressed in this article are solely those of the authors and do not necessarily represent those of their affiliated organizations, or those of the publisher, the editors and the reviewers. Any product that may be evaluated in this article, or claim that may be made by its manufacturer, is not guaranteed or endorsed by the publisher.
